# Undifferentiated Malignant Neoplasm Involving Parotid and Thyroid: Sampling and PAX8 Cross-Reactivity Can Obscure the Diagnosis of Lymphoma

**DOI:** 10.1155/2016/3291549

**Published:** 2016-12-18

**Authors:** Elizabeth W. Hubbard, Laurentia Nodit, Stuart Van Meter

**Affiliations:** Department of Pathology, University of Tennessee Graduate School of Medicine, Knoxville, TN, USA

## Abstract

Poorly differentiated malignant neoplasia arising within the head and neck region may originate from diverse sources. We report a case of a cytologically undifferentiated malignant neoplasm clinically presenting as masses involving thyroid and parotid. Although PAX8 was immunoreactive and thus worrisome for anaplastic thyroid carcinoma, the tumor was eventually proven to represent PAX5 positive diffuse large B-cell lymphoma expressing cross-reactivity with polyclonal PAX8 antibody. Cross-reactivity between commercially available polyclonal PAX8 and PAX5 immunostains has been described in the literature but is not widely known, and it is a potential pitfall for making a misdiagnosis. This distinction can have importance in selection of subsequent clinical therapy and should be considered in choice of immunohistochemical stains for diagnostic purposes.

## 1. Introduction

Poorly differentiated malignant neoplasms may be diagnostically challenging, especially in the head and neck region. Familiarity with the developmental embryology of head and neck anatomical structures as well as the unique molecular expression of common malignancies is necessary in interpreting immunohistochemistry (IHC) to pinpoint a specific diagnosis when confronted with an undifferentiated tumor. Knowledge of specific immunostain molecular targets and cross-reactivity of selected antibodies can be critical to avoid misinterpretation of overlapping immunoreactivity in unrelated diagnostic entities.

PAX8 is a transcription factor belonging to the paired-box gene family with a unique role in tissue development and limited expression in adult tissue, typically thyroid, thymic, renal, and nonmucinous Mullerian tissues [1]. Its utility in undifferentiated malignant neoplasms can be significant in the head and neck, where anaplastic thyroid carcinoma (ATC) can assume a variety of histologic patterns ranging from squamoid to focal papillary or more differentiated follicular to completely undifferentiated architecture [2]. Furthermore, ATCs often lose immunoexpression of more differentiated thyroid markers such as TTF-1 and thyroglobulin but retain PAX8 expression [3].

Original reports in the literature suggested PAX8 was also expressed in lymphoid cells [4]. More recent reports have noted cross-reactivity of commercially available polyclonal PAX8 immunostain antibodies with another paired-box transcription factor, PAX5 [5]. PAX5 is critical to the differentiation of B lymphocytes and is used clinically to detect B lymphocyte lineage, typically in lymphomas. Herein we present a case of a 71-year-old woman presenting with poorly differentiated thyroid and parotid masses initially interpreted as PAX8-immunoreactive but subsequently determined to be cross-reactive with PAX5, changing the diagnosis and therapeutic options.

## 2. Case Report

A 71-year-old woman presented with a three-week history of worsening shortness of breath and dysphagia. Past medical history was significant for hypothyroidism with long term thyroid replacement therapy, COPD, GERD, and hyperlipidemia. Outside hospital records revealed a 2.6 cm left parotid mass and markedly enlarged thyroid gland with circumferential narrowing of the trachea and extensive substernal extension. The patient had been treated there with intravenous steroids with respiratory status improvement.

**Figure 1:**
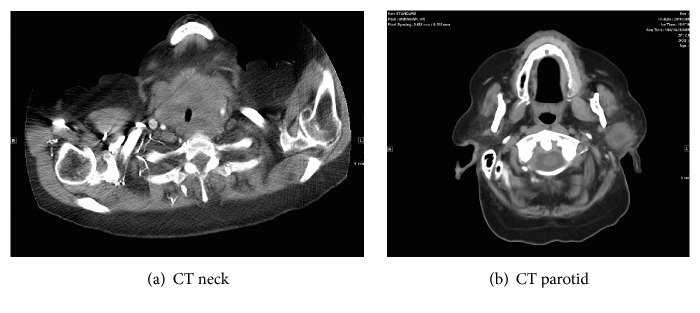
CT images of neck mass (a) demonstrating diffuse thyroid mass with tracheal luminal compression and left parotid mass (b) with central necrosis.

Physical examination revealed middle ear aerated bilaterally, no purulent secretions of the nose, and unremarkable throat exam. A markedly enlarged diffuse thyroid mass was noted extending below the clavicles. CT images (Figure 1) revealed a diffuse thyroid mass extending substernally with tracheal luminal compression to 7 mm, a left necrotic 2.6 cm parotid mass extending into the deep lobe, multiple pulmonary nodules, a pancreatic head mass, and possible serosal implants along the transverse colon. Overall, the findings were concerning for metastatic disease.

**Figure 2:**
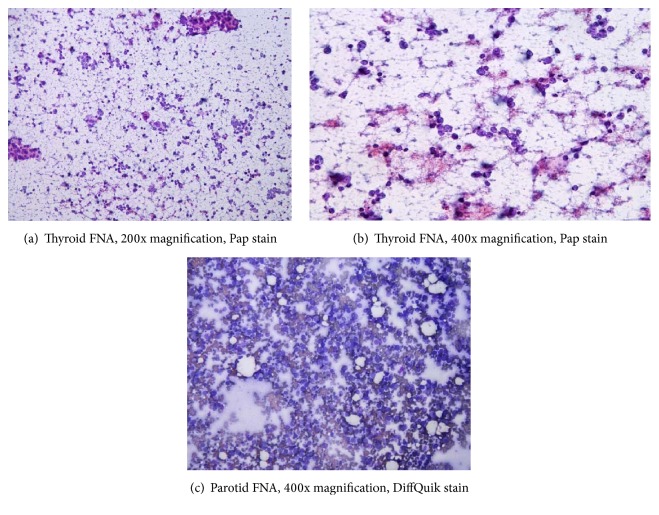
FNA of thyroid and parotid contained similar cytologic material with malignant cells arranged singly and in dyshesive groups in a necrotic background. A few background Hurthle cells are noted (a).

Ultrasound-guided fine needle aspiration was obtained from parotid and thyroid masses with preparation of a cell block from parotid mass material as well. Aspirate smears from thyroid and parotid masses appeared similar, composed of poorly differentiated cytologically malignant cells present in dyshesive groups and singly, having enlarged, vesicular nuclei with a thin rim of inconspicuous cytoplasm (Figures 2(a), 2(b), and 2(c)). Background necrosis and groups of infiltrating neutrophils were present. Within the thyroid aspirate, several groups of more cohesive cells were noted with abundant eosinophilic granular cytoplasm and round, regular nuclei, consistent with Hurthle cells (Figure 2(a)). In other areas, atypical cells formed tridimensional groups suggestive of possible thyroid follicle formation.

Flow cytometric immunophenotyping was performed on aspirate samples from the thyroid and parotid lesions. Samples were suboptimal, of low viability with limited events available for examination and therefore inconclusive, with 88% of cells failing to express lymphoid marker CD45. However, a small population of CD19+ CD20+ CD5− CD10− CD23− sIg kappa+ cells was detected. The specimen was interpreted as having a minute cellular population suspicious for involvement by a B-cell non-Hodgkin lymphoma involving 1% of the sample.

**Figure 3:**
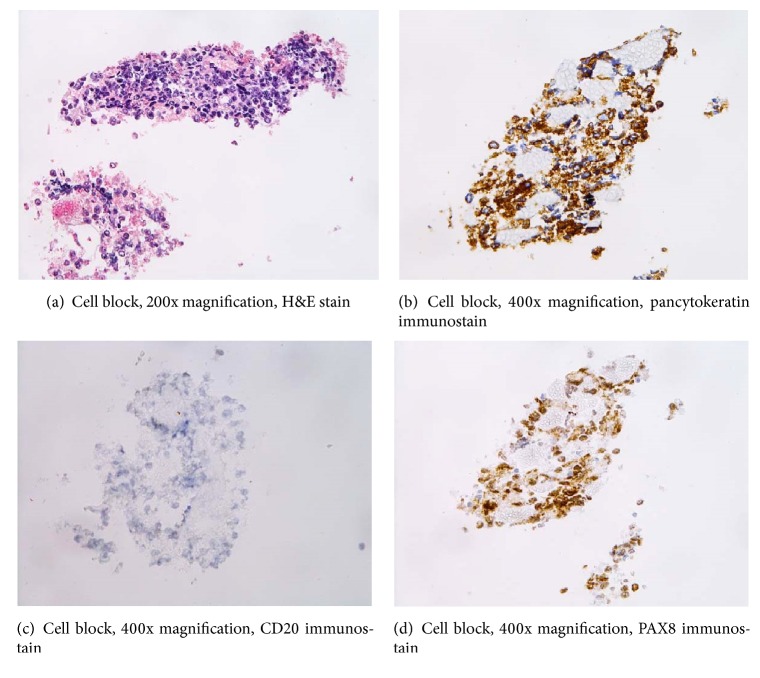
Cell block contained malignant cells expressing pancytokeratin cytoplasmic immunoreactivity and PAX8 nuclear staining but stained negatively with B lymphocyte marker CD20.

Cell block specimen from the parotid aspirate was quantitatively limited and contained cytologically malignant single cells in occasional aggregates remarkable for enlarged, vesicular nuclei with prominent nucleoli and scant cytoplasm in a necrotic background with red blood cells and scattered lymphoid cells (Figure 3(a)). IHC of malignant cells was interpreted as positive for pancytokeratin and PAX8 and negative for TTF-1, CD20, and CD3 (Figures 3(b), 3(c), and 3(d)). The diagnosis of poorly differentiated malignant neoplasm, favor carcinoma, was rendered. Anaplastic thyroid carcinoma was a consideration. Given the clinical presence of pancreatic and lung nodules, it was felt that a neuroendocrine carcinoma was unlikely but not excluded. Additional biopsy material was requested for precise classification.

**Figure 4:**
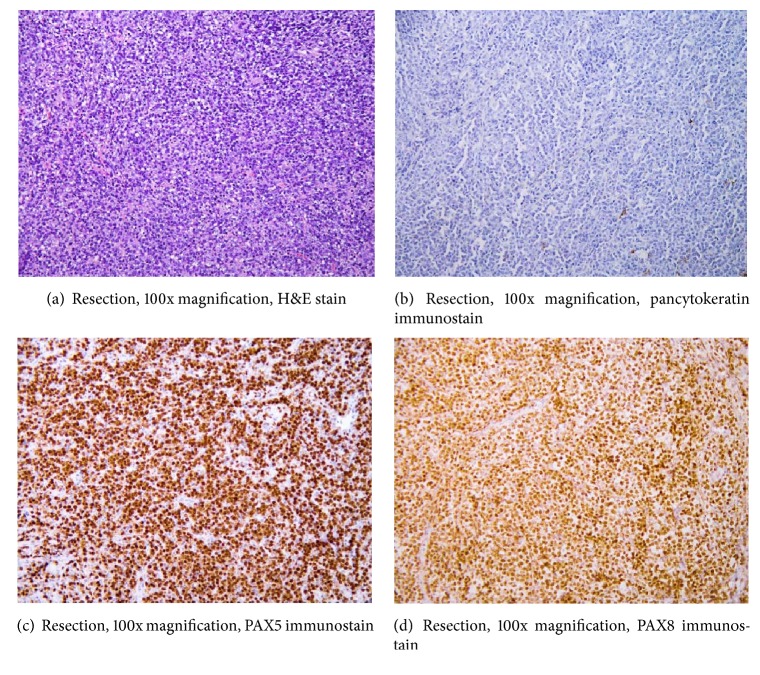
Isthmusectomy specimen demonstrates sheets of malignant cells displacing thyroid follicles; neoplasm is clearly pancytokeratin-negative with coexpression of PAX5 and polyclonal PAX8 immunoreactivity.

Owing to the clinical need for airway relief, an isthmusectomy and nodal tissue sampling was performed yielding multiple portions of pink-tan fibrotic tissue weighing 45 grams and measuring 6.5 cm in greatest aggregate dimension. No grossly recognizable thyroid parenchyma was identified. Microscopic sections revealed thyroid, skeletal muscle, and adipose tissue extensively infiltrated by a high grade malignant neoplasm composed of mononuclear cells with atypical, round to ovoid nuclei, vesicular chromatin, and prominent nucleoli (Figure 4(a)).

Brisk mitotic activity and apoptosis were present. Broad areas of confluent tumor necrosis were apparent. A few small islands of recognizable thyroid follicular epithelium with Hurthle cell change were entrapped within the mass and focally associated with neoplastic cells. Malignant cells were immunoreactive with CD45, CD20, PAX5, and PAX8 but failed to express pancytokeratin, CAM5.2, chromogranin, or synaptophysin (Figures 4(b), 4(c), and 4(d)). Rearrangement of MYC gene and *t*(8; 14) was detected by FISH without rearrangement of BCL2 or BCL6. Ki-67 proliferation rate was greater than 80%. EBV in situ hybridization was negative. The diagnosis of high grade CD20+ large B-cell lymphoma consistent with diffuse large B-cell lymphoma (DLBCL) was made.

## 3. Discussion

Undifferentiated malignancies can present a diagnostic challenge particularly when specimens are necrotic or quantitatively limited. Reliance on IHC requires specific knowledge of limitations of antibodies utilized. This case demonstrates a pitfall utilizing PAX8.

PAX8 is a transcription factor member of the paired-box gene family consisting of nine members, PAX1 through PAX9. Each transcription factor possesses a unique role in embryologic development and limited expression in adult tissue, although they share structural homology.* PAX* genes are further subdivided into subgroups on the basis of structural similarity with Subgroup II including PAX2, PAX5, and PAX8 [6]. Although initially described as a sensitive and specific marker for tumors of renal, Mullerian, or thyroid origin in both primary and metastatic sites, PAX8 immunoreactivity was observed occurring in all of 65 lymphoid tissues examined [4], primarily within lymphoid follicles and in parafollicular areas, thought to represent B lymphocytes. Other authors observed PAX8 immunoreactivity in diffuse large B-cell lymphoma (DLBCL) [7, 8]. Subsequently Morgan et al. reported reactivity of polyclonal PAX8 antibody (pPAX8) in nonneoplastic B cells and mature B-cell neoplasms, but not with monoclonal PAX8 (mPAX8) antibody within these tissues, and proposed cross-reactivity of the polyclonal commercially available PAX8 antibody with B-cell marker PAX5 [5]. They further cautioned that the diagnosis of B-cell lymphoma should be considered in cases of polyclonal PAX8 positive and epithelial marker negative neoplasia of unknown primary origin. Moretti et al. [9] demonstrated that N-terminal but not C-terminal antibody to PAX8 by IHC was present in reactive and neoplastic B lymphoid cells yet PAX8 mRNA levels were not detectable in any B-cell lymphoma cell lines studied by qRT-PCR methods. They concluded that PAX8 immunoreactivity reported in B-cell lymphomas was due to cross-reactivity of N-terminal regions of PAX5 and PAX8 due to highly conserved (70%) sequence homology. Further report by Conant et al. [10] showed that PAX8 N-terminal nuclear staining correlated with PAX5 staining in a series of B-cell neoplasms reviewed while PAX8 C-terminal staining was negative. They concluded that PAX8C has a role in discriminating between epithelial and lymphoid neoplasia but PAX8N, polyclonal PAX8, and PAX2 do not.

Our case was deceptive since the initial FNA cell block of parotid and thyroid masses was interpreted as pancytokeratin immunoreactive without expression of lymphoid markers CD3 or CD20. TTF-1−/PAX8+ expression in the limited cytologic material present suggested ATC. The presence of a minute CD20+ cellular population by flow cytometry was perplexing. While a single cell, dispersed cytologic architectural pattern is typical of lymphoma, ATC cells can occur singly or in dyshesive groups or intermixed with areas of better-differentiated thyroid carcinoma. TL can also be pleomorphic with dispersed cells arranged into tissue fragments and may be indistinguishable from ATC by light microscopy [11]. While the cell block material did contain several cellular groups and single cells, the material was nonetheless quantitatively limited and ultimately did not lead to a specific diagnosis, similar to the experience of others reported in the literature [12]. We initially attributed the pancytokeratin immunoreactivity to nonspecific uptake by nonviable malignant cells; however, in retrospect this was due to entrapped Hurthle cells within the neoplasm. Hurthle cells within the resection specimen were uniformly immunoreactive for PAX8, in keeping with the findings reported by Nonaka et al. [13].

In a series of three cases, Limmer et al. noted the diagnostic difficulty of separating ATC and TL by clinical, imaging, and cytologic results [14]. In their series, all patients, like ours, were women in the eighth or ninth decade of life presenting with dysphagia, dyspnea, and tracheal shift. Imaging demonstrated a thyroid mass with infiltrative growth pattern, sometimes with mediastinal lymph node involvement. Each patient underwent initial FNA with indeterminate results, requiring subsequent open biopsy for definitive diagnosis. ATC has an exceedingly poor prognosis (median survival from diagnosis being a few months) and clinical therapy may involve radical resection if surgically feasible, [15] followed by chemotherapy and radiation therapy. It is imperative to distinguish cases of TL, for which surgery may be avoided and prognosis can be excellent depending on the histologic subtype. They emphasized the importance of obtaining adequate viable tissue for precise diagnosis [14].

In summary, we present a case of a diffusely infiltrative poorly differentiated neoplasm presenting in the thyroid and parotid of a 71-year-old woman with dysphagia and dyspnea. Initial FNA diagnosis of a poorly differentiated malignant neoplasm was confused by initial interpretation of cell block PAX8 immunoreactivity with flow cytometry highlighting a minor population of B cells. Subsequent isthmusectomy confirmed DLBCL, with coexpression of PAX5 and polyclonal PAX8. This case highlights a pitfall possible with commercially available polyclonal PAX8 immunostains and the need for adequate viable tissue to discriminate between two potentially clinically and cytologically similar entities with very different prognosis and therapy.
